# Comparing Web-Based Venues to Recruit Gay, Bisexual, and Other Cisgender Men Who Have Sex With Men to a Large HIV Prevention Service in Brazil: Evaluation Study

**DOI:** 10.2196/33309

**Published:** 2022-08-04

**Authors:** Daniel Rodrigues Barros Bezerra, Cristina Moreira Jalil, Emilia Moreira Jalil, Lara Esteves Coelho, Eduardo Carvalheira Netto, Josias Freitas, Laylla Monteiro, Toni Santos, Cleo Souza, Brenda Hoagland, Valdilea Gonçalves Veloso, Beatriz Grinsztejn, Sandra Wagner Cardoso, Thiago Silva Torres

**Affiliations:** 1 Fundação Oswaldo Cruz Instituto Nacional de Infectologia Evandro Chagas Rio de Janeiro Brazil

**Keywords:** social media, web-based recruitment strategies, men who have sex with men, pre-exposure prophylaxis, PrEP, HIV prevention, Brazil, Latin America, HIV

## Abstract

**Background:**

Internet and mobile phones, widely available in Brazil, could be used to disseminate information about HIV prevention and to recruit gay, bisexual, and other cisgender men who have sex with men (MSM) to HIV prevention services. Data evaluating the characteristics of MSM recruited through different web-based strategies and estimating their cost and yield in the country are not available.

**Objective:**

We aimed to describe a web-based recruitment cascade, compare the characteristics of MSM recruited to a large HIV prevention service in Rio de Janeiro according to web-based venues, and estimate the cost per participant for each strategy.

**Methods:**

We promoted advertisements on geosocial networking (GSN) apps (Hornet and Grindr) and social media (Facebook and Instagram) from March 2018 to October 2019. The advertisements invited viewers to contact a peer educator to schedule a visit at the HIV prevention service. Performance of web-based recruitment cascade was based on how many MSM (1) were reached by the advertisement, (2) contacted the peer educator, and (3) attended the service. We used chi-square tests to compare MSM recruited through GSN apps and social media. The estimated advertisement cost to recruit a participant was calculated by dividing total advertisement costs by number of participants who attended the service or initiated preexposure prophylaxis (PrEP).

**Results:**

Advertisement reached 1,477,344 individuals; 1270 MSM contacted the peer educator (86 contacts per 100,000 views)—564 (44.4%), 401 (31.6%) and 305 (24.0%)—through social media, Grindr, and Hornet. Among the 1270 individuals who contacted the peer educator, 36.3% (n=461) attended the service with similar proportion for each web-based strategy (social media: 203/564, 36.0%; Grindr: 152/401, 37.9%; and Hornet: 107/305, 35.1%). MSM recruited through GSN apps were older (mean age 30 years vs 26 years; *P*<.001), more frequently self-reported as White (111/247, 44.9% vs 62/191, 32.5%; *P*=.03), and had higher schooling level (postsecondary: 157/254, 61.8% vs 94/194, 48.5%; *P*=.007) than MSM recruited through social media. GSN apps recruited MSM with higher HIV risk as measured by PrEP eligibility (207/239, 86.6% vs 133/185, 71.9%; *P*<.001) compared with social media, but there was no difference in PrEP uptake between the two strategies (*P*=.22). The estimated advertisement costs per participant attending the HIV prevention service were US $28.36 for GSN apps and US $12.17 for social media. The estimated advertisement costs per participant engaging on PrEP were US $58.77 for GSN apps and US $27.75 for social media.

**Conclusions:**

Social media and GSN app advertisements were useful to disseminate information on HIV prevention strategies and to recruit MSM to a large HIV prevention service in Brazil. Compared to GSN apps, social media advertisements were less expensive and reached more vulnerable and younger MSM. Digital marketing campaigns should use different and complementary web-based venues to reach a plurality of MSM.

## Introduction

Gay, bisexual, and other cisgender men who have sex with men (MSM) are disproportionately affected by HIV infection in Brazil [1], with HIV prevalence estimated at 18.4% [2]. Recent data point to increased prevalence of HIV among young MSM aged 18-24 years [3]. Since 2017, Brazil has been offering oral preexposure prophylaxis (PrEP) at no cost to individuals eligible for PrEP, including MSM [4]. However, increasing awareness of PrEP and other HIV prevention strategies among most vulnerable MSM, including young and those with lower income, remains a challenge [5].

Rio de Janeiro metropolitan area has more than 13 million inhabitants, the 16th largest urban area in the world [6]. Rio de Janeiro has a disproportionately large number of vulnerable individuals who live on the outskirts of the city. These social inequalities have great consequences for health access [7], including inadequate HIV care and prevention [8], which were intensified after the onset of the COVID-19 pandemic [9]. Among MSM from Rio de Janeiro, HIV prevalence was estimated at 15.3% in 2016 [2] and increased among those aged 18-24 years from 4.4% to 13.3% between 2009 and 2016 [3].

Internet and mobile phones are widely available in Brazil and could be used to disseminate information about HIV prevention services. Despite large inequalities, Brazil has a large number of internet users in all social strata: 74% of individuals receiving a minimum monthly wage (US $220.00) have access to the internet [10]. In Southeast Brazil, where Rio de Janeiro is located, 91% of the population have a mobile phone [11], and 99% have access to internet or apps via mobile phones [12].

Social media and geosocial networking (GSN) apps for sexual encounters (eg, Grindr, Hornet, and Scruff) are popular among MSM in Brazil [5,9,13,14]. A meta-analysis including 25 studies conducted in Australia, China, India, Thailand, and the United States showed that app users may have a higher prevalence of sexually transmitted infections (STIs) than nonusers [15]. In a web-based survey enrolling 11,367 Brazilian MSM, 93% reported using apps to seek sex partners, 54% of them with daily use. Willingness to use PrEP was similar among different sources of web-based recruitment, including GSN apps (Grindr and Hornet) and social media (Facebook and Instagram) [13].

Although different web-based venues have been used to recruit MSM, there are no Brazilian data evaluating the characteristics of MSM recruited through these strategies and estimating their cost and yield in the country. In this paper, we aimed to (1) describe the web-based MSM recruitment cascade, (2) compare the characteristics of MSM recruited to a large HIV prevention service according to different web-based venues, and (3) estimate the cost per individual of each strategy.

## Methods

### Study Design

We performed a digital marketing campaign targeting MSM from March 2018 to October 2019 using advertisements on GSN apps (Hornet and Grindr) and social media (Facebook and Instagram) to increase HIV knowledge, HIV testing, and PrEP awareness. The Instituto Nacional de Infectologia Evandro Chagas, Fundação Oswaldo Cruz (INI-Fiocruz) study team designed and implemented the campaign [16]. INI-Fiocruz has the largest HIV prevention service for adults (18 years or older) in Rio de Janeiro city and its metropolitan area in Brazil. The service is part of the Brazilian Public Health System (*Sistema Único de Saúde* [SUS]) at no cost to the user.

### Recruitment Strategies

Hornet and Grindr are popular among MSM in Brazil, and recruitment using these GSN apps has been used in previous cross-sectional studies conducted in the country [5,9,13,14,17,18]. We used direct inbox messages and banners to advertise on Hornet and Grindr, respectively. In addition, we created posts on social media (Facebook and Instagram), which were boosted and appeared at user’s feeds. We targeted social media users interested in subjects related to LGBTQIA+ people (people of diverse genders and sexualities, inclusive of and not limited to lesbian, gay, bisexual, transgender, queer, intersex, asexual, questioning, and pansexual) communities. Advertisement messages were built during meetings with key members of MSM communities from different socioeconomic status and race. These advertisements had information about HIV prevention and invited viewers to contact a peer educator (via phone number, email, and WhatsApp) to schedule a visit at the HIV prevention service for HIV risk assessment, HIV testing and referral to PrEP or postexposure prophylaxis (PEP).

### Variables

All MSM referred by the peer educator who attended the HIV prevention service answered semistructured interviews conducted by trained counselors. Covariables were age at the time of the visit (categorized in 18-24, 25-35, and >35 years); race (Black, Pardo or mixed, and White); schooling (elementary [≤9 years], secondary [10-12 years] and postsecondary [>12 years]) and Municipal Human Development Index, a 3-level dimension measure of human development (life expectancy, education, and income), dichotomized into very high (≥0.800) or other (<0.800) [19].

HIV testing was offered to all individuals who attended the HIV prevention service. HIV status was dichotomized into negative and positive according to HIV rapid test results, following Brazilian recommendations [20]. Counselors evaluated all HIV-negative MSM for PEP and PrEP eligibility based on Brazilian National Guidelines. Individuals reporting condomless sex in the previous 72 hours were referred for PEP [21]. MSM with at least one of the following criteria in the last 6 months were eligible for PrEP: (1) condomless receptive anal sex, (2) sex with partner living with HIV, (3) transactional sex (in exchange for money, goods, and benefits, among others), and (4) history of STI [22]. PrEP uptake was defined as the number of participants who initiated PrEP divided by the number of participants eligible for PrEP [23].

### Data Analyses

We evaluated the web-based recruitment cascade for Hornet, Grindr, and social media based on the number of MSM who (1) viewed the advertisement; (2) contacted the peer educator; and (3) attended the HIV prevention service. Conversion rate was calculated dividing the number of MSM who contacted the peer educator by those reached by advertisements. We used chi-square test to compare the characteristics of MSM recruited through dating apps (Hornet and Grindr) with those of MSM recruited by social media (Facebook and Instagram). We estimated the advertisement costs (in US dollars) to recruit one participant to HIV prevention service by dividing total advertisement costs by the number of individuals who attended the service (GSN apps or social media). Lastly, we used the same rationale to estimate the cost per individuals engaging on PrEP. All analyses were conducted in R Studio, using R version 4.0.3 (R Foundation for Statistical Computing).

### Ethical Considerations

This study was reviewed and approved by the INI-Fiocruz institutional review board (#CAAE 26095519.1.0000.5262). The board waived the informed consent as data were collected retrospectively from participants’ charts and exams collected at INI-Fiocruz during HIV testing or prevention routine. Most participants attended the service only once, and personal information such as telephone number or address were nonexistent or outdated, avoiding collection of informed consent a posteriori.

## Results

**Figure 1:**
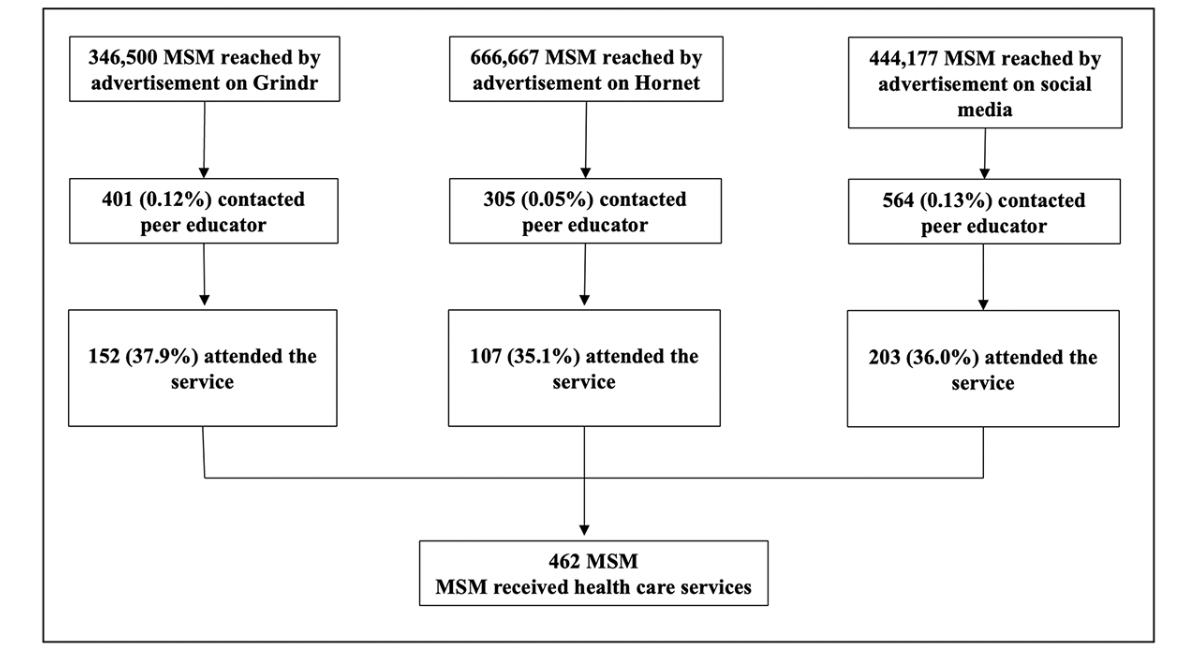
Web-based cascade to recruit gay, bisexual, and other cisgender men who have sex with men (MSM) for a large HIV prevention service at Rio de Janeiro, Brazil.

Our marketing campaign (all strategies combined) reached 1,477,344 individuals—346,500 (23.8%) from Grindr, 666,667 (45.7%) from Hornet, and 444,177 (30.5%) from social media (Figure 1). Overall, 1270 MSM contacted the peer educator (86.0 contacts per 100,000 views), with a higher conversion rate on social media (564/444,177, 0.13%) compared to Grindr (401/346,500, 0.12%) and Hornet (305/666,667, 0.05%). Among the 1270 MSM contacting the peer educator, 564 (44.4%), 401 (31.6%), and 305 (24.0%) were recruited on social media, Grindr, and Hornet, respectively. Of all 1270 MSM who contacted the peer educator, 36.3% (n=462) attended the HIV prevention service, with similar proportions for each web-based strategy (Grindr: 152/401, 37.9%; Hornet: 107/305, 35.1%; and social media: 203/564, 36.0%).

MSM attending the HIV prevention service (N=462) had a median age of 28 years (IQR 23-34), mostly self-identified as Black or Pardo (265/438, 60.5%), completed postsecondary schooling (251/448, 56.0%), and lived in very high Municipal Human Development Index neighborhood (301/461, 65.3%). A total of 38 (8.2%) MSM tested positive for HIV. Among the 424 HIV-negative MSM, 340 (80.2%) were eligible for PrEP; PrEP uptake was 62.9% (214/340). Only 22 (5.2%) MSM were eligible for and initiated PEP.

MSM recruited by GSN apps were older (mean age 30 years vs 26 years; *P*<.001) and reported White race (111/247, 44.9% vs 62/191, 32.5%; *P*=.03) and higher schooling level (postsecondary: 157/254, 61.8% vs 94/194, 48.5%; *P*=.007) than MSM from social media (Table 1). MSM from GSN apps reported higher HIV risk as measured by PrEP eligibility (207/239, 86.6% vs 133/185, 71.9%; *P*<.001) than MSM from social media, but there was no difference on PrEP uptake (*P*=.22).

The estimated advertisement cost per participant attending the HIV prevention service was US $28.36 for GSN apps and US $12.17 for social media. The estimated advertisement cost per participant engaging in PrEP was US $58.77 for GSN apps and US $27.75 for social media.

**Table 1:** Characteristics of gay, bisexual, and other cisgender men who have sex with men (MSM) recruited to HIV prevention service according to web-based strategies.

Characteristics		Total (N=462)	GSN^a^ apps (n=259)	Social media (n=203)	*P* value	
Age (years), median (IQR)		28 (23-34)	30 (24-37)	26 (23-31)	<.001	
**Age range (years), mean (SD)**						
	18-24	146 (31.7)	66 (25.5)	80 (39.8)	<.001	
	25-35	210 (45.7)	116 (44.8)	94 (46.8)		
	>35	104 (22.6)	77 (29.7)	27 (13.4)		
**Race, n (%)**						.03
	White	173 (39.5)	111 (44.9)	62 (32.5)		
	Black	87 (19.9)	43 (17.4)	44 (23.0)		
	Pardo or Mixed	178 (40.6)	93 (37.7)	85 (44.5)		
**Schooling, n (%)**						.007
	Elementary	10 (2.2)	7 (2.8)	3 (1.5)		
	Secondary	187 (41.7)	90 (35.4)	97 (50)		
	Postsecondary	251 (56.0)	157 (61.8)	94 (48.5)		
**MHDI^b^, n (%)**						.57
	Very high	301 (65.3)	172 (66.4)	129 (63.9)		
	Other	160 (34.7)	87 (33.6)	73 (36.1)		
**HIV status, n (%)**						.66
	Negative	424 (91.8)	239 (92.3)	185 (91.1)		
	Positive	38 (8.2)	20 (7.7)	18 (8.9)		
**PEP^c^ initiation (n=424), n (%)**						
	Yes	22 (5.2)	11 (4.6)	11 (5.9)	.54	
	No	402 (94.8)	228 (95.4)	174 (94.1)		
**PrEP^d^ eligibility (n=424), n (%)**						<.001
	Yes	340 (80.2)	207 (86.6)	133 (71.9)		
	No	84 (19.8)	32 (13.4)	52 (28.1)		
**PrEP uptake (n=340), n (%)**						.22
	Yes	214 (62.9)	125 (60.4)	89 (66.9)		
	No	126 (37.1)	82 (39.6)	44 (33.1)		

^a^GSN: geosocial networking.

^b^MHDI: Municipal Human Development Index.

^c^PEP: postexposure prophylaxis.

^d^PrEP: preexposure prophylaxis.

## Discussion

### Principal Results

Our digital marketing campaign on different web-based venues was effective in recruiting MSM to a large HIV prevention service in Brazil. We successfully reached and engaged diverse MSM of different ages and races and high PrEP eligibility. Nevertheless, MSM with lower education was the least accessed group, indicating that complementary recruitment strategies such as those on LGBTQIA+ venues or respondent-driven sampling [24] may still be necessary to reach MSM at higher social vulnerability.

Social media recruited a larger proportion of young MSM, with lower income and schooling compared to those recruited via GSN apps. This underscores the importance of using social media to recruit MSM for HIV prevention services and to promote prevention campaigns targeting young MSM at HIV risk in Rio de Janeiro, Brazil. In addition, social media were more cost-effective to recruit individuals to attend the HIV prevention service and to use PrEP. Facebook was the most cost-effective web-based venue to recruit MSM to a qualitative study in Seattle [25] and was effective to recruit Latino gay couples in a study conducted in New York [26]. These findings confirm that social media platforms such as Facebook and Instagram could be used as an alternative option to reach and engage MSM in other HIV prevention services in resource-constrained settings such as Brazil.

By contrast, a study conducted in Philadelphia identified Grindr as the most effective strategy to recruit people for an HIV vaccine trial [27]. Although PrEP uptake did not differ between individuals recruited by different strategies, GSN apps were more effective in recruiting high-risk MSM, according to PrEP eligibility, compared to social media. As such, different web-based venues may be useful to recruit diverse MSM to HIV prevention services, especially young MSM, as shown in previous studies conducted in the United States [27,28].

### Strengths

In addition to recruitment to the HIV prevention service, our campaign aimed to increase PrEP awareness, although the latter could not be measured in this study. Data from web-based surveys showed an increase in PrEP awareness in Rio de Janeiro, Brazil, from 2016 to 2018 [29], and 2020 national data indicated that 87% of sexual and gender minorities recruited on Hornet were aware of PrEP [9]. Moreover, HIV knowledge was associated with PrEP use among MSM eligible for PrEP in Brazil [17]. Digital campaigns are of utmost importance to promote information on HIV prevention, including PrEP and other prevention technologies, among MSM. These results are particularly useful to develop public strategies and may contribute to designing campaigns to increase PrEP uptake in Brazil. Lastly, our study was conducted before the COVID-19 pandemic, and due to the increased use of digital recruitment for scientific studies [30], a more heterogeneous population would be expected to be reached nowadays.

### Limitations

This study considered only MSM reached by a digital marketing campaign who contacted the peer educator. The advertisements may have reached other MSM who attended the HIV prevention service without contacting the peer educator or who attended other services in Rio de Janeiro, Brazil. Responses of web-based recruitment and PrEP or PEP eligibility were self-reported, thus introducing the possibility of recall, response, or social desirability bias. Participants reported sexual behavior during risk assessment for PrEP or PEP evaluation. However, data on sexual behavior practices have not been systematically collected, preventing further associations between web-based recruitment and sexual practices (eg, condomless receptive sex or transactional sex). This study helped to identify this issue, leading to modifications aiming to collect more detailed data on sexual behavior and other variables, such as substance use and previous STIs. Lastly, venue-based recruitment and peer referral may perform better than web-based recruitment for most vulnerable MSM (young, Black or Pardo, low-income, and low-educated individuals) [16], highlighting the importance of community networks.

### Conclusions

Advertisements on social media and GSN apps were useful to disseminate information on HIV prevention strategies and to recruit MSM to an HIV prevention service in Brazil. Compared to GSN apps, social media advertisements were less expensive and reached more vulnerable and younger MSM. Our findings indicate that digital marketing campaigns should use different and complementary digital venues to reach diverse MSM.

## Abbreviations

GSN: geosocial networking
HDI: human development index
INI-Fiocruz: Instituto Nacional de Infectologia Evandro Chagas, Fundação Oswaldo Cruz
LGBTQIA+: people of diverse genders and sexualities, inclusive of and not limited to lesbian, gay, bisexual, transgender, queer, intersex, asexual, questioning, and pansexual
MSM: gay, bisexual, and other cisgender men who have sex with men
PEP: postexposure prophylaxis
PrEP: preexposure prophylaxis
STI: sexually transmitted infection
